# Intratumoral microbiota in orchestrating cancer immunotherapy response

**DOI:** 10.1515/jtim-2024-0038

**Published:** 2025-01-10

**Authors:** Yutian Zou, Hanqi Zhang, Feng Liu, Zhe-Sheng Chen, Hailin Tang

**Affiliations:** State Key Laboratory of Oncology in South China, Guangdong Provincial Clinical Research Center for Cancer, Sun Yat-sen University Cancer Center, Guangzhou, 510060, Guangdong Province, China; Department of Cancer Research Institute, Hengyang Medical School, University of South China, Hengyang 421001, Hunan Province, China; College of Pharmacy and Health Sciences, St. John’s University, NY 11439, New York USA

## Introduction

**Figure 1:**
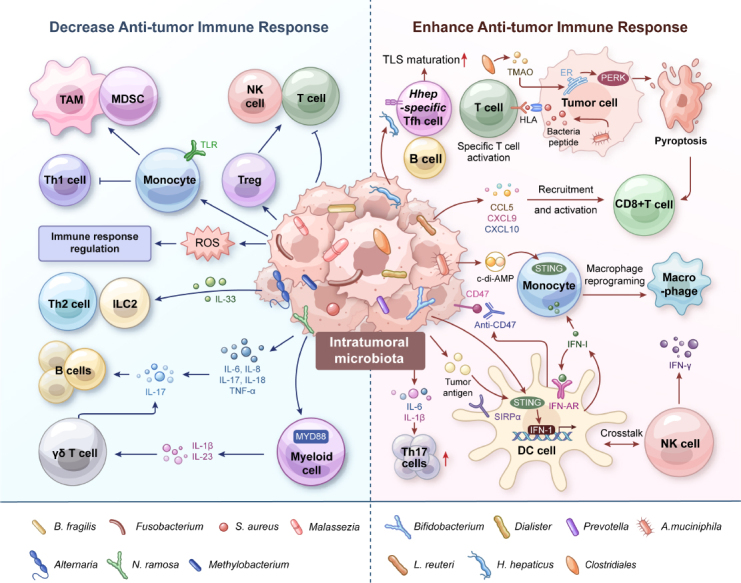
Mechanisms of intratumoral microbiota in orchestrating cancer immune response. Intratumoral microbiota can either enhance anti-tumor immunity (*e.g*., *via* STING activation, T/NK cell activation, antigen presentation) or suppress anti-tumor immunity (*e.g*., anti-inflammatory response, ROS production, T cell inhibition).

The intratumoral microbiota is a dynamic ecosystem of bacteria, fungi, and viruses in tumor tissues, and it is increasingly recognized as a significant factor in cancer progression and therapeutic outcomes. Emerging research highlights the dual impact of the intratumoral microbiota on anti-tumor immunity, similar to the gut microbiome.^[1,2]^ Specific microbial communities can activate immune responses, driving inflammation and promoting cytotoxic immune cell infiltration, enhancing the efficacy of immunotherapy. In contrast, other microbial populations can create an immunosuppressive environment that is not conducive to tumor immunotherapy. Understanding these mechanisms is vital for improving current therapies and developing new strategies. The complex interplay between microbiota, the tumor microenvironment, and the host immune system offers potential for personalized, more effective cancer immunotherapy. The complex interplay between microbiota, the tumor microenvironment, and the host immune system offers potential for personalized, more effective cancer immunotherapy (Figure 1).

## The promotive role of intratumoral microbiota in anti-tumor immunity

Intratumoral microbiota might modulate anti-tumor immunity in many ways, including activating the STING and PERK pathways, promoting lymph node maturation, and enhancing cellular immunity mediated by bacterial peptides (References and citations are displayed in Supplementary Table 1). Shi *et al*. showed that treating *Bifidobacterium* by oral route promotes anti-CD47 immunotherapy through the STING pathway in mice. *Bifidobacterium* activated the STING pathway in dendritic cells, thereby enhancing the production of type I interferon for further stimulation of anti-tumor responses mediated by CD8^+^ T cells in combination with anti-CD47 therapy. Moreover, *Akkermansia muciniphila* contributes to the interferon (IFN)-I-natural killer axis by induction the STING pathway by producing cyclic dimeric adenosine monophosphate (di-AMP). Such activation releases IFN-I, chemokines CXCL10 and CCL5, and cytokines interleukin-15 (IL-15)/IL-15R and IL-18, thereby allowing crosstalk of NK cells and dendritic cells for reprogramming of macrophages for an improved therapeutic outcome in melanoma patients.

Beyond STING pathway activation, intratumoral bacteria can regulate other immune pathways. In triple-negative breast cancer, *Clostridiales*-derived trimethylamine oxide (TMAO) , also an important metabolite within the gut microbiota, induces tumor cell pyroptosis via the PERK pathway.^[3]^ This process, characterized by increased cleaved caspase-3 and gasdermin E (GSDME), activates CD8+ T cell-mediated anti-tumor immunity. Combining intratumoral TMAO injection with systemic anti-PD-1 therapy further enhances anti-tumor effects macroscopically. Moreover, intratumoral bacteria can influence the formation of lymphoid structures. In *Helicobacter hepaticus*-colonized colorectal cancer (CRC), increased T follicular helper (Tfh) cells promoted the maturation of tertiary lymphoid structures (TLS) near the tumor, enhancing lymphatic transport and activating Tfh cells. This resulted in significant shrinkage of the tumor in *H. hepaticus*-colonized mice. Lastly, tumor immunity is contributed to through bacterial peptide presentation. Kalaora *et al*. identified immunogenic bacterial peptides derived from *Staphylococcus caprae* and *Actinomyces odontolyticus* within melanoma cells. These peptides, presented on MHC class I and II molecules, activate T-cell responses, pointing out bacterial peptide presentation’s role in stimulating cellular immunity.

## The suppressive role of intratumoral microbiota in anti-tumor immunity

Despite these beneficial roles, the intratumoral microbiota can also hinder anti-tumor immunity and reduce cancer immunotherapy efficacy (Table 1). Targeting these detrimental components offers a strategy to enhance treatment responses. When mucosal barriers are compromised, *Bacteroides fragilis* and *Fusobacterium* drive intestinal tumorigenesis by generating reactive oxygen species (ROS), which regulate localized inflammation but can impair T cell function and anti-tumor responses.

Similarly, *Nevskia ramosa* and *S. aureus* promote regulatory T cell (Treg) activation, facilitating prostate and liver cancer progression. Additionally, *Aspergillus sydowii* enhances lung tumor growth by IL-1β-dependent activation of myeloid-derived suppressor cells, which inhibit cytotoxic T cell function and increase PD-1-expressing CD8+ T cells.

Chronic antigen exposure and a microbial metabolite-augmented immunosuppressive milieu can lead to T cell exhaustion, characterized by loss of effector function and increased PD-1 expression. This exhausted state reduces T cell responsiveness to immunotherapies like checkpoint inhibitors. In pancreatic ductal adenocarcinoma (PDAC), the microbiome promotes immune tolerance by modulating tumor-associated macrophages (TAMs) *via* TLR signaling, fostering an immunosuppressive environment.^[4]^ Recent studies show that PDAC-derived tryptophan metabolites activate the aryl hydrocarbon receptor (AhR) in macrophages, promoting tumor-supportive polarization, reducing inflammatory T-cell infiltration, and enhancing PDAC growth.^[5]^

The intratumoral microbiota can induce anti-inflammatory properties and T-cell dysfunction, reducing anti-tumor immune responses. In PDAC, intratumoral fungi stimulate IL-33 secretion, recruiting T helper 2 (Th2) and innate lymphoid cells (ILC2). Antifungal therapy has been shown to contribute to PDAC regression, offering a novel treatment strategy. *Fusobacterium nucleatum* binds CRC cells via FadA, inducing IL-6, IL-8, and IL-18, while *Escherichia coli* virulence factor CNF1 triggers IL-6 and TNF-α, sustaining chronic inflammation that promotes tumor progression.^[6]^ Intratumoral bacteria also stimulate myeloid cells to produce MyD88-dependent IL-1β and IL-23, activating γδT cells to secrete IL-17, which promotes B cell infiltration and tumor progression. In breast cancer, *F. nucleatum* correlates negatively with CD3+ T cell infiltration, inhibiting T-cell aggregation and promoting TAM and fibroblast recruitment.^[7]^ A new tellurium-containing polycarbonate with cisplatin (PTE@ CDDP) inhibits *F. nucleatum* by disrupting its membrane integrity, reducing its load in colorectal cancer, and reversing inflammation and intestinal barrier damage. PTE@CDDP enhances cisplatin bioavailability, enables ROS-responsive drug release, and overcomes chemotherapy resistance. This antimicrobial polymer-based drug delivery approach offers a promising strategy for synergistic immunotherapy by modulating the tumor microenvironment and mitigating microbial-induced immunosuppression.^[8]^

## Future perspectives in targeting the intratumoral microbiota

The interplay between intratumoral microbiota and cancer immunotherapy offers new therapeutic strategies. Future strategies could involve using targeted antibiotics, engineered probiotics, RNA vaccines, or bacteria as potential approaches to precisely modulate the tumor microenvironment.^[9]^ Advanced delivery systems, like nanoparticles triggered by microbial signals, may enhance precision.^[10]^ Identifying microbiome-based biomarkers and targeting microbial-host interactions could refine immune responses. Artificial intelligence may further clarify complex microbiome-tumor-immune interactions. Addressing specificity and off-target effects across tumor heterogeneity is essential to realize the potential of microbiome-based cancer therapies.

### Supplementary Information

Supplementary materials are only available at the official site of the journal (www.intern-med.com).

## Supplementary Material

Supplementary Material
