# Supplementary material for: Intratumoral microbiota in orchestrating cancer immunotherapy response
Source: J Transl Int Med. 2025 Jan 10;12(6):540–2. doi: 10.1515/jtim-2024-0038 (PMC11720933; doi:10.1515/jtim-2024-0038)
Supplement: Supplementary file 1 — Supplementary Material [file jtim-2024-0038_sm.pdf]

Supplementary Table 1: Intratumoral microbiota and their impact on cancer immunotherapy efficacy

Function to anti-tumor immune response	Microbiota type	Tumor type	Target cell	Main pathway or molecule	Mechanism	Reference
Enhance	*Bifidobacterium*	Breast cancer	Monocyte, Dendritic cell	STING pathway	Activating dendritic cells *via* the STING signaling pathway	[1]
	A.muciniphila	Melanoma	NK cell, Dendritic cell	STING pathway	Promoting antigen presentation	[2]
	Clostridiales	Breast cancer	$CD8^{+}$ T-cell	PERK pathway	Related metabolite trimethylamine N-oxide induced pyroptosis in tumor cells by activating the endoplasmic reticulum stress	[3]

					kinase PERK and thus enhanced $CD8^+$ T cell-mediated anti-tumor immunity in TNBC *in vivo*	
	S. caprae, A. odontolyticus	Melanoma	T cell	HLA-bound peptides	Deriving immunogenic HLA-I and HLA-II bacterial peptides and activating cellular immunity after being presented to T cells	[4]
	H. hepaticus	Colorectal cancer	Tfh cell	TLS	Increasing the number of Tfh cells in the colon and promoting the maturation of the TLS adjacent to the tumor	[5]
Decrease	*F. nucleatum*	Colorectal cancer	Cancer cell	TLR4 and MYD88 pathway	Targeting TLR4 and MYD88 innate immune signaling and specific microRNAs to	[6]

				activate the autophagy pathway	
B. fragilis	Colorectal cancer	Macrophage	NLRP3 pathway	Inhibiting macrophage activation and reducing the release of proinflammatory cytokines such as IL-18 and IL-1β	[7]
Malassezia *Alternaria*	Pancreatic ductal adenocarcinoma;	TH2 and innate lymphoid cells 2	KRAS pathway	Facilitating IL-33 secretion	[8]
Fusobacterium	Pancreatic ductal adenocarcinoma;	Myeloid cell	ROS	Increasing production of ROS and inflammatory cytokines	[9]
Methylobacterium	Gastric cancer	$CD8^+$ T cell	TGF-β pathway	Inhibiting immune infiltration of $CD8^+$ T cells	[10]
N. ramosa	Prostate cancer		immunosuppression	Inducing inflammation, promoting immunosuppression	[11]

A.muciniphila: *Akkermansia muciniphila*; *S. caprae*: *Staphylococcus caprae*; *A. odontolyticus*: *Actinomyces odontolyticus; H. hepaticus*: *Helicobacter*

hepaticus; F. nucleatum: *Fusobacterium nucleatum; B. fragilis*: *Bacteroides fragilis; N. ramosa*: *Nevskia ramose*; Tfh. T follicular helper; TLS: tertiary lymphoid structures; ROS: reactive oxygen species; IL: interleukin; TGF-β: transforming growth factor-β.

REFERENCES

1. Shi Y, Zheng W, Yang K, Harris KG, Ni K, Xue L, *et al*. Intratumoral accumulation of gut microbiota facilitates CD47-based immunotherapy *via* STING signaling. J Exp Med 2020;217(5):e20192282.

2. Lam KC, Araya RE, Huang A, Chen Q, Di Modica M, Rodrigues RR, *et al*. Microbiota triggers STING-type I IFN-dependent monocyte reprogramming of the tumor microenvironment. Cell 2021;184(21):5338–5356.e21.

3. Wang H, Rong X, Zhao G, Zhou Y, Xiao Y, Ma D, *et al*. The microbial metabolite trimethylamine N-oxide promotes antitumor immunity in triple-negative breast cancer. Cell Metab 2022;34(4):581–594.e8.

4. Kalaora S, Nagler A, Nejman D, Alon M, Barbolin C, Barnea E, *et al*. Identification of bacteria-derived HLA-bound peptides in melanoma. Nature 2021;592(7852):138–143.

5. Overacre-Delgoffe AE, Bumgarner HJ, Cillo AR, Burr AHP, Tometich JT, Bhattacharjee A, *et al*. Microbiota-specific T follicular helper cells drive tertiary lymphoid structures and anti-tumor immunity against colorectal cancer. Immunity 2021;54(12):2812–2824.e4.

6. Liang P, Chen Q, Chen X, Zhang X, Xiao Y, Liang G, *et al*. Microbiota modulate immune repertories in lung adenocarcinoma *via* microbiota-immunity interactive network. Transl Lung Cancer Res 2024;13(10):2683–2697.

7. Shao X, Sun S, Zhou Y, Wang H, Yu Y, Hu T, *et al*. Bacteroides fragilis restricts colitis-associated cancer *via* negative regulation of the NLRP3 axis. Cancer Lett 2021;523:170–181.

8. Alam A, Levanduski E, Denz P, Villavicencio HS, Bhatta M, Alhorebi L, *et al*. Fungal mycobiome drives IL-33 secretion and type 2 immunity in

pancreatic cancer. Cancer Cell 2022;40(2):153–167.e11.

9. Wei MY, Shi S, Liang C, Meng QC, Hua J, Zhang YY, *et al*. The microbiota and microbiome in pancreatic cancer: more influential than expected. Mol Cancer 2019;18(1):97.

10. Peng R, Liu S, You W, Huang Y, Hu C, Gao Y, *et al*. Gastric microbiome alterations are associated with decreased $CD8^+$ tissue-resident memory T cells in the tumor microenvironment of gastric cancer. Cancer Immunol Res 2022;10(10):1224–1240.

11. Ma J, Gnanasekar A, Lee A, Li WT, Haas M, Wang-Rodriguez J, *et al*. Influence of intratumor microbiome on clinical outcome and immune processes in prostate cancer. Cancers 2020;12(9):2524.
